# Physical activity and creativity of children and youths

**DOI:** 10.1186/s12887-020-2017-2

**Published:** 2020-03-12

**Authors:** Nitita Piya-amornphan, Anoma Santiworakul, Salila Cetthakrikul, Phatcharawadee Srirug

**Affiliations:** Department of Physical Therapy, School of Allied Health Sciences, Walailak University, Nakhon Si Thammarat, 80160 Thailand

**Keywords:** Childhood cognitive function, Creativity, Intelligence, Physical activity

## Abstract

**Background:**

Childhood is an important period for developing maturity in thinking. Accumulating evidence shows the association between physical activity and cognitive function. Although both the intelligence quotient and emotional quotient have been reported to be associated with physical activity, there is a limited amount of published research regarding the association between physical activity and cognitive function in children and youths. With respect to creativity, an important skill for the twenty-first century, little evidence on the creative quotient promotion in childhood is available. The present study, therefore, is designed to explore the correlation between physical activity and creativity.

**Methods:**

The participants included 1447 students with different age groups in 34 schools from Southern Thailand. Age groups were categorized according to Thailand’s 2016 Report Card on Physical Activity for Children and Youth, where 521 students were aged 6–9 years, 487 students were aged 10–13 years, and 439 students were aged 14–17 years. Creativity was measured through the use of the Test for Creative Thinking-Drawing Production (TCT-DP). Active play, time with family and peers, and sedentary behavior were monitored by the Thailand Physical Activity Children Survey-the Student Questionnaire (TPACS-SQ).

**Results:**

The correlation between the TCT-DP score representing creativity and active play was noticed in adolescents (*r* = 0.148, *p* = 0.001), but not found in participants aged 6–13 years. Active play was associated with time with family and peers in all age groups (*r* = 0.485, *p* <  0.001).

**Conclusions:**

The present data supports the idea that optimal physical activity is required during childhood for developing thinking process. Promotion of active play with family and peers may facilitate creativity skills.

## Background

Physical activity is known to affect health outcomes throughout one’s life [1, 2]. The optimal level of physical activity can improve health status and reduce the incidence of chronic diseases (e.g. cardiovascular disease, diabetes, cancer, and depression) [2, 3]. The World Health Organization recommends that adults should perform aerobic physical activity for at least 150 min at moderate intensity or 75 min at vigorous intensity throughout the week, whereas children and youths should accumulate at least 60 min of moderate to vigorous intensity physical activity daily [3]. In children and youths, the nine common indicators related to physical activity have been identified by the global matrix of grades. The indicators include the following five behaviors: 1) overall physical activity, 2) organized sport participation, 3) active play, 4) active transportation, and 5) sedentary behavior; and the following four key influences: 1) family and peers, 2) school, 3) community and built environment, and 4) government strategies and investments [4]. Literature showed that active play, sedentary behavior, and time with family and peers have been frequently studied. Physical activity has been markedly achieved through active play, active play was reported to increase physical activity and prevent childhood obesity [5, 6]. Family and peers provide the social support for promoting physical activity, it includes situations and conditions in which parents/peers facilitate physical activity for their children/friends [7]. Family and peers have a strong impact on physical activity during childhood. There is evidence showing that parenting practices are associated with how children prefer to play [7, 8], peer relationships enhance an engagement in team sports and other physical and leisure activities as well [9, 10]. Sedentary behavior has been classified as behavior that falls under the screen-time guidelines [11]. In contrast to indicators potentiating physical activity (i.e. active play and time with family and peers), sedentary behavior is a global health risk leading to an increased rate of premature death, especially from non-communicable diseases [11]. Although active play, time with family and peers, and sedentary behavior have significantly affected physical activity in children and youths, the association among these indicators are still elusive. Whether they interact with each other should be clarified.

Besides health improvement and disease prevention, physical activity among young people is important for growth and development [11, 12]. Regarding cognitive function, brain function and academic achievement have been reported to be associated with physical activity [13–15]. The association between physical activity, healthiness, and the intelligence quotients of 135 high school students in Jeddah in the Kingdom of Saudi Arabia was reported, and the findings indicated that the intelligence quotient was positively associated with physical activity and health status [16]. In addition, there is evidence that demonstrates a positive link between the emotional quotient and physical activity in 599 Taiwanese college students [17]. Higher emotional quotient was also associated with longer duration of exercise (weekly hours) in 64 participants with a mean aged of 19.88 years [18]. Taken together, these cross-sectional studies revealed that an increase in physical activity was associated with both higher intelligence quotient and emotional quotient in childhood.

Critical thinking, communication, collaboration, and creativity have been classified as the most prominent twenty-first century competencies identified on the basis of making a measurable contribution to educational accomplishments, relationships, employment, and health and well-being outcomes [19]. Creativity has been defined as the ability to produce novel and appropriate work. *Novel* work means original or unexpected, while *appropriate* work implies useful or adaptive [20, 21]. Creativity is important for social development because that lead to driving innovation and responding to unforeseen problems [19]. Cognitive abilities attributed to prefrontal cortex function are required for creativity. In humans, the prefrontal cortex does not completely mature until the early 20s [20, 21]. Childhood, thus, is a valuable time for development of creative skills. Information showing how to promote creativity development according to cognitive milestones is required. The objectives of the present study therefore aimed at exploring the correlation between physical activity and creativity in children and youths. Indicators of physical activity including active play, time with family and peers, sedentary behavior, and their relevant associations were also areas of focus in this study.

## Methods

### Stratified sampling

The sample size in Thailand’s 2016 Report Card on Physical Activity for Children and Youth was calculated based on the number of students across the country, and a multistage stratified cluster sampling was adopted to recruit students into the study. Thailand geographically is divided into 9 regions with 77 provinces and 878 districts. All provinces within each region were stratified according to their population size. Each province was then randomly recruited; the city districts were purposively selected and other districts were randomly chosen. Schools were finally randomly enlisted, herein the response rate was approximately 84.3% of those invited. Students within each school were then classified by gender and age, and they were proportionally recruited according to the size of the school. Participants were divided into three age groups, which included 6–9, 10–13, and 14–17 years of age [11]. These age groups were mainly classified by the age structure and education in Thailand. The classification reflected the differences in physical and cognitive development milestones among each age group. All healthy students were included in the study. Skeletal or other diseases affecting physical activity had not been reported in each student. Height, weight, and body mass index were screened in all students before data collection. In the present study, data was collected from 1447 students in 34 schools from Southern Thailand. These students completed responses to both the physical activity questionnaire and creativity test. There were a negligible number of students declining the participation.

### Physical activity survey

The data set of physical activity in the present study is the same set reported in Thailand’s 2016 Report Card on Physical Activity for Children and Youth. Physical activity of students was assessed by using the Thailand Physical Activity Children Survey-the Student Questionnaire (TPACS-SQ). TPACS-SQ was developed for each age group in order to accommodate the differences in student capability and maturity classified by the age structure and education in Thailand. All healthy students were asked the number of days that they retained activity/behavior as recommended per day over the past 7 days, and a list of activities such as play, sports, recreation, and leisure activity was provided to students in all age groups as an example. For students aged 6 to 9 years, face-to-face interviews were conducted within a class by using image cards. In the other two older age groups, the students completed the survey in a class facilitated by three research team members and a class teacher. Test-retest reliability of the items measuring physical activity was reported in the study of Amornsriwatanakul et al. in 2016 [11].

### Creativity assessment

**Fig. 1:**
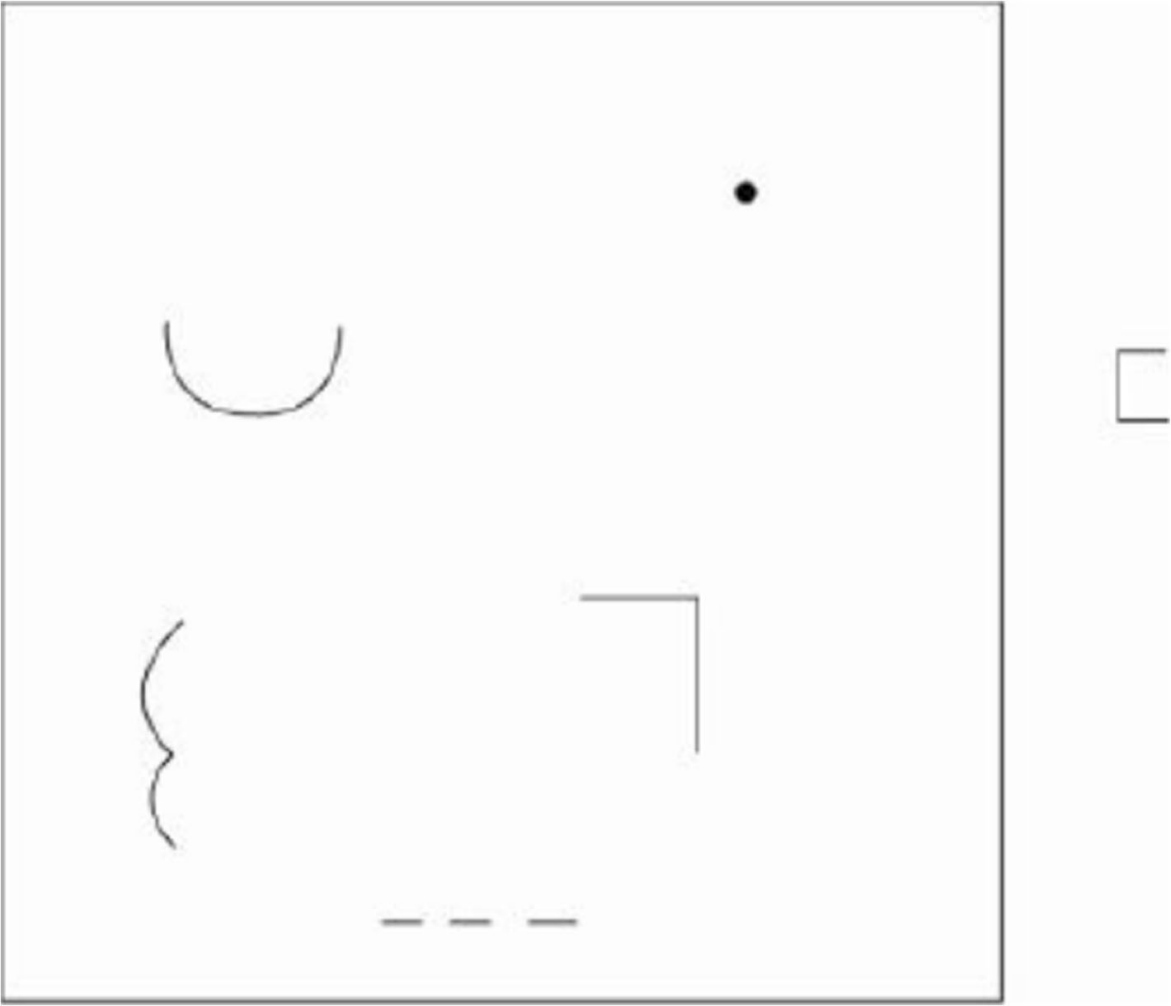
A form of TCT-DP, Urban and Jellen (1989)

Creativity was determined in the present study through the use of the Test for Creative Thinking-Drawing Production (TCT-DP) carried out following the protocol of Urban reported in 2004 [22, 23]. The reliability and validity of TCT-DP has been provided in many studies [22, 23], and it has been previously reported that experience in drawing is unrelated to the TCT-DP score [23]. When finishing a physical activity survey, the same set of research team members informed the students how to do the test. Participants were asked to complete a drawing with six flawed elements placed on a creativity test sheet© Jellen and Urban (1989) (Fig. 1) [24], all students performed this test autonomously and by themselves. An assessment of the TCT-DP consisted of fourteen criteria which include the following: 1) continuations, 2) completions, 3) new elements, 4) connections made with a line, 5) connections that contribute to a theme, 6) boundary breaking that is fragment-dependent, 7) boundary breaking that is fragment-independent, 8) perspective, 9) humor and affectivity, 10) unconventionality with, manipulation of the test material, 11) unconventionality with, abstract elements, 12) unconventionality in the use of symbols, 13) unconventionality with, unconventional usage of the given fragments, and 14) speed [23]. These creativity scores were independently assessed by three research team members in which the final TCT-DP result was the sum of the points obtained in all tested criteria. All creativity results were double-checked by the lead author.

### Statistical analysis

Data of the physical activity survey were carried out by using CSPro V6.1 (U.S. Census Bureau). In the present study, all analyses were conducted through using SPSS V21. Kruskal-Wallis test was used to determine the difference of physical activity indicators and creativity among age groups. Correlation analyses was then performed with the Spearman’s correlation. Association between physical activity indicators, including active play, time with family and peers, and sedentary behavior, and creativity was primarily monitored. Correlation among these physical activity indicators was also ascertained. The statistical significance level was *p*-value ≤0.05. The Spearman correlation coefficient generally indicated weak, moderate, or strong association between variables. Regression analysis was also performed to determine the impact of physical activity indicator on creativity score.

## Results

### Participant characteristics

All healthy students were included in the present study in which health status was screened through their health report obtained from a class teacher. No health problem was reported for all students. Skeletal or other diseases affecting physical activity had not been informed in each student, impairment of motor and cognitive functions was also not noticed. Height, weight, and body mass index of all participants were in normal range. The data from 1447 students were categorized into three age groups, which included 6–9 years, 10–13 years, and 14–17 years based on the age structure and education in Thailand (Supplementary Table 1). The number of students in each group ranged from 439 to 521. There were no markedly differences in gender proportion among any of the groups (Supplementary Table 1).

### Physical activity pattern and the creativity score

With respect to the TPACS-SQ and guideline for physical activity in children and youths, any day with at least 60 min of active play was recorded, and it was found that children and youths spent between 3 and 4 days per week on average in recommended active play. In addition to active play, spending over 2 h per day with family and peers was determined; the results of the survey indicated that participants did this around 2 days per week on average. When measuring sedentary behavior, the findings indicated that participants engaged in sedentary behavior that was not more than 2 h per day for about 3 to 4 days per week on average. Duration patterns of physical activity indicators in each age group were similar. They spent the most amount of time in sedentary behavior followed by active play followed by the least amount of time being spent with family and peers (Table 1). Adolescents were the most sedentary (Table 1). The total TCT-DP scores which indicated creativity ability in the present study ranged between 9 and 69 points, and the TCT-DP score increased in accordance with the age group (Table 1).

**Table 1:** Baseline of physical activity and creativity

Parameters	6–9 years *n* = 521	10–13 years *n* = 487	14–17 years *n* = 439	*p*–value
Physical activity indicator				
Active play at least 60 min (day/week)	3 (0–7)	3 (0–7)	3 (0–7)	0.173
Family and peers at least 2 h (day/week)	2 (0–7)	2 (0–7)	2 (0–7)	0.172
Sedentary behavior not more than 2 h (day/week)	3 (0–7)	3 (0–7)	2 (0–7)	0.039^*^
TCT-DP	20 (10–46)	21 (9–43)	24 (11–69)	< 0.001^***^

Data expressed as median (min–max), * *p*-value ≤ 0.05; *** *p*-value ≤ 0.001

### Correlation between physical activity indicators and creativity

**Fig. 2:**
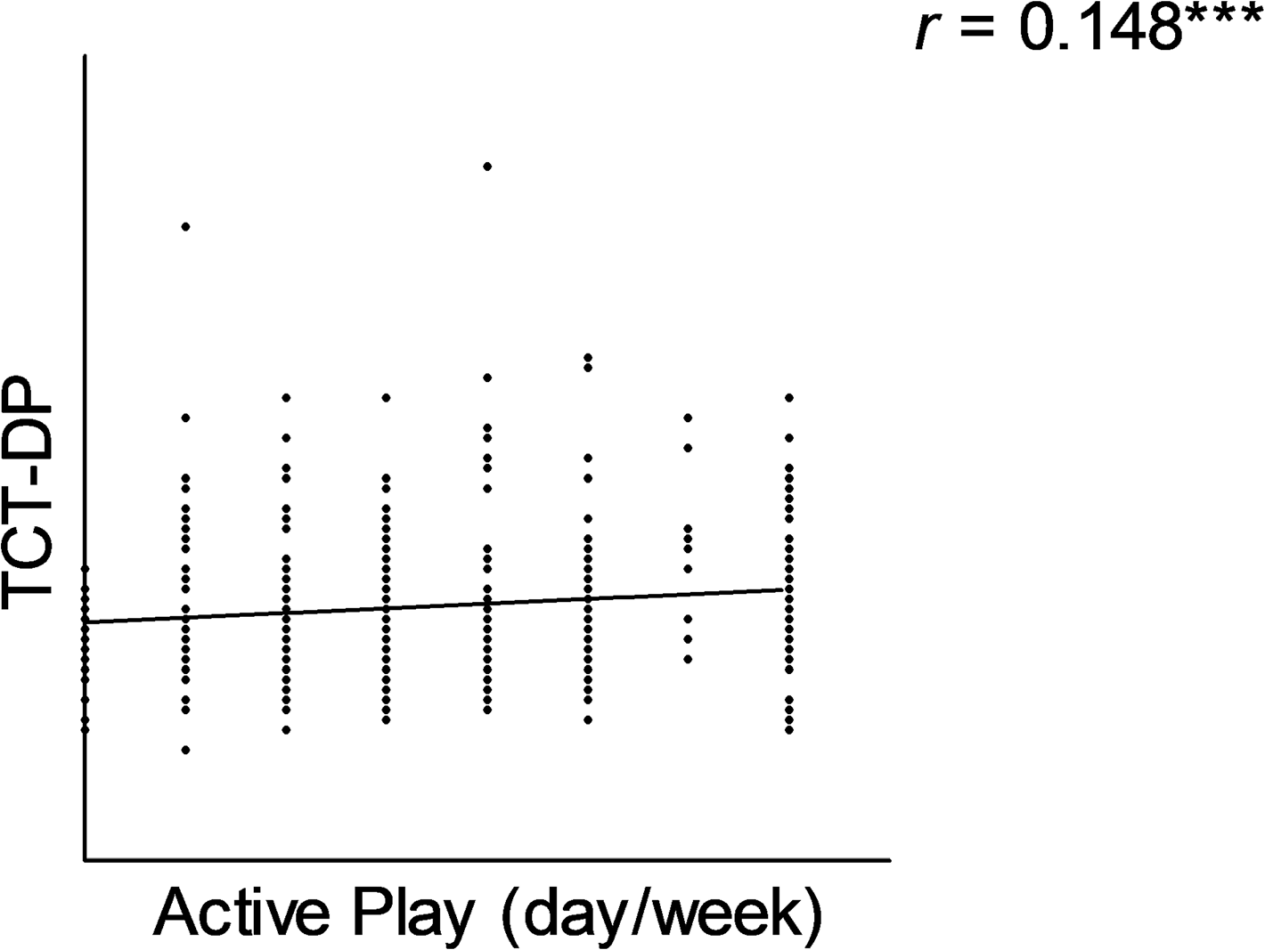
Correlation between active play and creativity

In children aged 6–9 years and 10–13 years, the TCT-DP score was not associated with all three indicators of physical activity (Table 2). The correlation between creativity and active play was present in participants aged 14–17 years (Fig. 2). Impact of active play on creativity was analyzed by using regression analysis. It was showed that TCT-DP was not depended on active play (Supplementary Table 2).

**Table 2:** Physical activity indicators and creativity

Physical activity indicator	TCT–DP		
	6–9 years *n* = 521	10–13 years *n* = 487	14–17 years *n* = 439
Active play			
Correlation coefficient (*r*)	0.010	0.014	0.148***
*p*–value	0.408	0.379	0.001
Family and peers			
Correlation coefficient (*r*)	0.056	0.020	0.040
*p*–value	0.099	0.327	0.204
Sedentary behavior			
Correlation coefficient (*r*)	−0.034	0.056	−0.050
*p*–value	0.217	0.108	0.150

*** *p*-value ≤ 0.001

### Association among the physical activity indicators of children and youths

Association among three physical activity indicators, including active play, time with family and peers, and sedentary behavior was observed in 1447 participants, aged 6–17 years. All indicators were associated with each other, the highest level of association was an association between active play and time with family and peers (Table 3).

**Table 3:** Correlation among the children and youth’s physical activity indicators

Physical activity indicator	Active play	Family and peers	Sedentary behavior
Active play			
Correlation coefficient (*r*)	1.000	0.485***	0.081**
*p*–value		< 0.001	0.002
Family and peers			
Correlation coefficient (*r*)	0.485***	1.000	0.088***
*p*–value	< 0.001		0.001
Sedentary behavior			
Correlation coefficient (*r*)	0.081**	0.088***	1.000
*p*–value	0.002	0.001	

*n* = 1447, ** *p*-value ≤ 0.01; *** *p*-value ≤ 0.001

## Discussion

Physical activity indicators reported in the present study partly conduced a grading of the physical activity indicators showed in Thailand’2016 Report Card [11]. It was found that children and youths had an active play grade of “F”, indicating that only 0–20% of them met the defined benchmark. At the same time, sedentary behavior of children and youths received a grade of “D–”, meaning that 21–25% of them met the optimal level. A better grade was given to the time spent with family and peers, in which 66–75% of children and youth met the guideline, resulting in a grade of “B” [11]. Taken together, Thai children and youths lowly participated in physical activity and active play, their sedentary behaviors are high. These results also reflect an increasing in physical inactivity and sedentary behavior among children and youths [11, 25]. Since physical inactivity and sedentary behavior have contributed to the global burden of various morbidities and premature mortality [26]. This incident in children and youths thus should be strongly concerned. In the present study, active play was found to be associated with time spent with family and peers in all age groups. It supports the idea that the family and peers is an important factor for enhancing physical activity in children and youths [7–10]. A strategy for increasing active play and time spent with family and peers combined with a lessening in sedentary behavior may fight against the problems of an increasing physical inactivity and sedentary behavior and support health promotion in children and youths.

The mean and range of the TCT-DP score of children and youths in the present study were in line with the previous studies [27–30], it also indicated that there was a limited number of students with high-creativity abilities. An increasing in the TCT-DP according to age group can be explained by the fact that the brain region involved in generating creative ideas does not mature until humans pass their 20th birthday [20, 21]. Differences in creative thought were discovered between different age groups, using functional magnetic resonance imaging while participants carried out divergent thinking tasks [31]. It was demonstrated that default and executive networks were more functionally coupled for creative process in older than younger adults [31]. Although age has been found to affect creativity, there is a creativity difference among age-matched individuals [32]. This fact supports the idea that a high creativity ability may associate with other factors affecting cognitive function. The present study thus aimed at exploring the correlation between physical activity indicators and creativity in children and youths. Creativity was determined through using TCT-DP which has been considered a reliable test [23]. However, Jankowska et al. [30]. reported that TCT-DP might also require a specific profile of gaze distribution or self-regulation strategies because the more time participants spent looking at the many pieces in the test sheet, the higher their scores were. The dwell time for a single fragment, in contrast, was negatively related to the TCT-DP score [30].

As mentioned previously, the optimal level of physical activity has been reported to improve cognitive function [1–3]. In childhood, physical activity is associated with the intelligence and emotional quotients [16–18]. Latorre Román et al. in 2017 [33], reported the association between physical fitness and creativity in elementary school children (*n* = 308, age from 8 to 12 years). The participants completed a fitness test battery and the Prueba de Imaginación Creativa para Niños (PIC-N; Creative Imagination Test for Children). It was observed that physical fitness was positively correlated with creativity, and boys had better physical fitness and higher creativity skill [33]. In the present study, the difference in creativity were not observed between genders. The association between creativity and physical activity indicators was specifically monitored according to age groups divided by the age structure and education in Thailand. The result thus reflects that association in each age group in which their cognitive and physical functions are different. In addition, this study pointed to the physical activity indicators, including active play, time with family and peers, and sedentary behavior which has been frequently reported to affect physical activity and physical fitness in children and youth [5–10]. The results showed that creativity was associated with active play in adolescents; however, the association was not found in participants aged 6–14 years. All results of physical activity indicators were collected by using a self-report instrument, recall ability has been reported as a weakness of the self-report instrument [11]. Recalling the types and amount of time spent in physical activity might be difficult for children, especially in the youngest age group [11]. Since active play has been reported as the directly indicator of physical activity in children and youths [5, 6]. The present study thus supports the finding that physical activity is essential for cognitive development in children and youths [13–15]. Increased physical activity, especially in active play may be a strategy for facilitating creativity. Nevertheless, the present results also implied that there are many factors related to creativity. To enhance creativity ability in this generation, more information is needed to clarify other factors associated with creativity.

### Limitations and future plan

Health status reported in the present study was observed only through health report obtained from a class teacher, but more demographic and health condition information should be considered for future studies as well. As physical activity is important for cognitive function and related with intelligence quotient, emotional quotient, and creativity skills in childhood. Future longitudinal study thus plan to investigate the difference of these variables in adolescents with low and high physical activity. An objective measurement of behaviors and a screening tool for assessing the time spent with family and peers should be consider included.

## Conclusions

Physical activity is important to good health, especially in childhood, which is the golden period for physical and mental development. Physical activity has been reported to enhance both the intelligence quotient and emotional quotient. The present study supports the positive correlation between physical activity and creativity ability; indeed, the active play indicator was dominant.

## Supplementary information

**Additional file 1 Supplementary Table 1.** Characteristics of participants. **Supplementary Table 2.** Impact of active play on creativity.

**Additional file 2.** Raw data.

## Data Availability

Not applicable in regard to the raw data of physical activity because the data was entered in a specific program (CSPro V6.1) and it is authorized by the Physical Activity Research Centre (PARC). The data represented in the present study, including active play, time spent with family and peers, sedentary behavior, and creativity scores are provided in an additional supporting file.

## Abbreviations

TCT-DP: Test for Creative Thinking-Drawing Production
TPACS-SQ: Thailand Physical Activity Children Survey-the Student Questionnaire
