# Supplementary material for: Physical activity and creativity of children and youths
Source: BMC Pediatr. 2020 Mar 12;20:118. doi: 10.1186/s12887-020-2017-2 (PMC7068971; doi:10.1186/s12887-020-2017-2)
Supplement: Supplementary file 1 — Additional file 1 Supplementary Table 1. Characteristics of participants. Supplementary Table 2. Impact of active play on creativity. [file 12887_2020_2017_MOESM1_ESM.docx]

**Supplementary Table 1** Characteristics of participants

| Characteristics | |
| --- | --- |
| 6-9 years  Mean age (years)  Educational level range  Number  Gender  Male  Female | 7.72 (1.09)  grade 1-4, elementary education  521  262 (50.29%)  259 (49.71%) |
| 10-13 years  Mean age (years)  Educational level range  Number  Male  Female | 11.38 (1.08)  grade 4-6, elementary education to grade 7, secondary education  487  249 (51.13%)  238 (48.87%) |
| 14-17 years  Mean age (years)  Educational level range  Number  Gender  Male  Female | 15.54 (1.12)  grade 7-12, secondary education  439  223 (50.80%)  216 (49.20%) |

**Data expressed as mean (SD), Basic education in Thailand has been divided into elementary education and secondary education. Prathom is namely grade in elementary education and Matthayom is namely grade in secondary education. There are Prathom 1-6 and Matthayom 1-6.**

**Supplementary Table 2** Impact of active play on creativity

| Model | Unstandardized coefficients | | Standardized coefficients | t | *p*-value |
| --- | --- | --- | --- | --- | --- |
|  | B | Std. error | Beta |  |  |
| (constant)  Active play | 24.308 | 1.098 |  | 22.132 | 0.000 |
|  | 0.418 | 0.271 | 0.074 | 1.541 | 0.124 |

**Dependent variable was TCT-DP.**
