# Supplementary material for: Physical activity and creativity of children and youths
Source: BMC Pediatr. 2020 Mar 12;20:118. doi: 10.1186/s12887-020-2017-2 (PMC7068971; doi:10.1186/s12887-020-2017-2)
Supplement: Supplementary file 2 — Additional file 2. Raw data. [file 12887_2020_2017_MOESM2_ESM.pdf]

gender	age	CQ	PA1	PA2	PA3
female	6	20	1	3	7
male	6	18	7	7	0
female	6	18	2	3	0
male	6	15	2	1	6
male	6	18	3	0	0
female	6	24	2	3	2
female	7	18	5	3	0
male	7	18	6	5	6
male	7	18	7	4	4
male	7	15	7	6	2
female	7	19	5	3	0
female	7	17	5	3	0

male	8	21	7	7	0
male	8	18	7	7	0
male	8	18	2	1	0
female	8	27	0	2	3
female	8	28	4	7	1
female	8	19	1	1	1
male	9	19	4	2	4
male	9	22	5	2	4
male	9	23	5	2	3
female	9	21	7	1	2
female	9	18	7	2	2
female	9	16	7	1	1
female	7	34	7	5	0
female	7	18	7	3	0
female	7	17	6	7	7

female	7	20	2	5	0
female	7	18	6	5	0
male	7	19	7	4	2
male	7	19	3	3	0
male	7	16	4	5	5
male	7	20	2	5	2
female	9	21	3	0	7
female	9	14	0	0	5
female	9	19	0	0	4
male	9	17	0	5	7
male	9	15	7	5	7
male	9	16	7	0	7
female	9	23	7	0	3
female	9	17	1	0	7
female	9	20	7	6	7

male	9	17	0	0	3
male	6	15	1	1	2
female	6	17	7	0	5
female	6	15	1	0	7
male	6	16	7	2	5
female	7	16	2	0	7
male	7	14	2	0	7
female	8	26	3	0	7
female	8	20	4	0	7
male	8	26	4	2	6
male	8	24	4	3	5
female	9	24	7	7	0
female	9	18	7	7	3
male	9	17	0	0	1
male	9	21	5	5	3

female	8	16	4	1	4
female	8	19	2	0	7
male	8	22	4	1	1
female	8	17	1	0	2
female	8	16	1	0	0
female	8	21	1	0	2
male	8	22	1	0	1
male	9	17	1	0	0
female	9	20	2	0	1
female	9	17	2	0	7
female	9	17	2	0	7
female	9	12	1	0	7
female	9	20	2	0	7
male	9	22	4	0	1
male	9	23	4	0	1

male	9	16	0	1	0
male	9	15	5	3	1
female	7	21	2	3	4
male	7	16	4	2	1
female	7	24	3	1	5
female	8	24	3	1	4
male	8	23	1	2	4
female	8	20	1	0	0
male	8	14	1	0	1
male	6	16	1	1	0
female	6	18	1	0	0
male	6	22	3	1	0
male	6	21	3	2	0
female	6	23	1	1	0
female	6	14	0	0	0

female	6	16	2	3	2
male	6	14	4	5	7
female	6	17	3	6	4
female	6	15	2	3	2
male	6	15	3	7	4
female	7	20	9	9	9
male	7	17	5	2	6
male	7	18	5	5	6
female	7	19	2	0	5
male	8	29	1	1	1
female	8	20	0	1	1
female	8	15	2	2	3
male	8	16	2	2	3
male	8	20	1	2	1
male	8	21	2	1	3

male	8	17	2	3	5
male	8	19	2	2	0
female	9	19	2	1	0
female	9	19	2	1	0
male	9	20	6	2	1
male	9	26	2	1	0
male	9	24	0	2	0
female	9	23	3	1	1
female	6	15	7	7	6
male	6	21	4	2	0
female	7	18	4	2	4
female	7	16	7	7	7
female	7	14	0	0	0
female	7	10	0	0	0
female	8	17	6	2	7

female	8	16	1	1	5
female	8	16	5	5	2
male	8	18	4	2	0
male	8	20	7	7	0
male	8	26	7	7	0
male	9	26	7	7	7
female	9	19	6	6	7
female	9	18	7	7	5
female	9	16	0	0	1
male	9	19	0	0	2
male	9	19	7	1	7
male	9	23	7	1	7
male	7	15	3	2	4
male	7	25	2	2	0
female	7	25	1	5	7

male	8	19	6	4	4
female	8	19	5	0	0
male	8	17	5	0	7
male	8	17	5	5	7
female	8	19	0	0	3
female	8	25	7	2	0
female	9	15	0	0	5
female	9	21	7	2	7
male	9	12	7	0	7
male	9	19	7	7	2
female	9	17	0	0	5
male	9	10	7	2	0
male	6	23	3	0	0
male	7	17	3	4	0
male	7	22	2	3	0

male	7	16	7	7	0
male	7	21	0	0	7
female	7	15	2	0	2
female	7	16	3	1	2
female	7	22	2	2	2
male	7	23	2	2	2
male	7	14	4	0	2
female	7	10	5	0	7
female	8	20	0	0	1
male	8	15	5	5	5
male	8	16	5	7	5
male	8	21	7	7	2
female	9	14	1	0	3
female	9	16	1	0	4
female	9	21	0	0	7

male	9	17	4	3	7
female	9	21	0	0	7
male	9	20	4	2	5
female	9	18	0	0	2
female	9	21	5	5	0
male	9	29	5	5	5
female	9	21	4	4	0
male	9	30	7	5	3
female	6	15	1	2	7
female	7	19	0	3	7
female	7	21	0	4	1
male	7	21	2	0	2
female	7	16	4	3	7
male	7	18	3	0	0
female	8	22	7	7	7

female	8	21	7	7	7
male	8	23	0	2	0
male	8	18	2	2	0
male	8	21	1	4	4
male	8	20	2	3	0
female	8	27	3	5	1
male	8	20	5	2	0
female	9	23	4	3	2
female	9	18	1	5	7
female	9	18	1	5	7
female	9	18	1	5	7
male	9	24	7	3	1
male	9	23	5	1	2
male	9	21	5	1	1
female	6	16	3	1	1

male	6	19	7	2	7
male	6	24	7	2	7
female	6	15	2	0	2
male	7	24	7	0	7
female	7	17	4	2	7
female	7	18	4	2	7
female	7	16	1	0	1
female	7	15	1	0	1
female	7	16	1	1	1
male	7	19	2	2	2
female	7	23	1	0	0
male	7	14	2	2	0
female	7	16	3	6	5
female	7	21	3	4	5
female	7	21	3	4	5

female	7	24	0	0	2
female	8	17	3	0	5
male	8	15	3	3	4
male	8	19	7	7	5
male	8	20	6	1	2
male	8	15	1	3	5
female	8	20	3	2	3
female	8	22	3	7	7
female	9	22	0	0	3
female	9	19	1	0	2
female	9	20	0	0	2
female	9	30	5	5	6
female	9	23	7	6	7
male	9	16	7	2	2
female	9	19	5	0	0

female	9	20	5	0	1
male	9	20	7	0	5
male	9	15	1	0	0
male	9	16	4	2	1
male	9	27	7	3	7
male	9	23	7	7	7
female	9	18	7	0	7
female	9	19	7	0	7
female	9	22	6	0	7
male	6	21	7	3	7
female	6	19	5	1	7
male	6	17	3	2	7
female	6	15	3	4	7
female	6	16	7	3	7
male	7	18	7	4	7

male	7	21	7	0	7
female	7	17	8	0	7
male	7	17	1	2	0
male	7	13	0	2	7
male	7	19	0	0	7
female	7	17	0	0	7
female	7	20	0	0	7
female	7	25	0	0	7
female	8	33	1	0	7
female	8	22	1	0	7
female	9	20	7	6	7
male	9	17	6	7	6
female	9	18	7	3	7
female	9	18	7	3	7
male	9	13	7	6	1

male	9	20	4	3	5
male	9	15	5	2	5
male	6	21	7	3	7
female	6	19	5	1	7
male	6	17	3	2	7
female	6	15	3	4	7
female	6	16	7	3	7
male	7	18	7	4	7
male	7	21	7	0	7
female	7	17	8	0	7
male	7	17	1	2	0
male	7	13	0	2	7
male	7	19	0	0	7
female	7	17	0	0	7
female	7	20	0	0	7

female	7	25	0	0	7
female	8	33	1	0	7
female	8	22	1	0	7
female	9	20	7	6	7
male	9	17	6	7	6
female	9	18	7	3	7
female	9	18	7	3	7
male	9	13	7	6	1
male	9	20	4	3	5
male	9	15	5	2	5
female	6	25	2	1	0
female	6	17	1	1	0
male	6	27	0	0	0
female	6	20	0	0	0
female	6	26	1	1	2

male	6	21	2	0	0
female	6	19	1	0	0
male	6	17	3	0	0
male	6	17	2	0	0
male	6	16	2	0	0
female	7	21	2	0	3
male	7	18	7	0	3
male	7	12	7	1	7
male	7	22	0	0	1
male	7	15	1	1	0
female	7	15	7	0	7
female	7	15	7	1	7
male	7	17	0	0	7
male	7	16	7	0	7
female	8	22	3	2	3

female	8	23	3	2	2
female	8	27	5	2	6
female	8	18	4	3	5
female	8	22	3	2	2
male	8	18	5	5	2
male	8	22	5	0	3
male	8	30	5	3	7
male	8	26	7	5	5
male	8	19	3	4	5
male	8	18	2	2	2
female	9	23	3	5	7
female	9	20	3	5	7
female	9	22	2	1	7
male	9	21	5	6	3
male	9	24	5	0	2

female	9	18	2	1	2
male	9	21	3	0	5
male	9	20	5	0	4
male	9	20	7	0	0
male	9	17	4	0	2
female	6	21	7	2	0
male	6	22	2	2	0
male	6	21	3	2	0
male	6	33	5	1	0
male	6	19	4	0	0
male	7	32	2	0	7
male	7	19	0	0	0
male	7	32	0	3	7
male	7	21	0	1	7
male	7	19	0	4	0

male	7	17	0	2	0
male	7	31	0	3	7
female	7	18	1	0	0
female	7	19	1	0	0
female	7	17	1	0	0
female	8	20	7	5	2
female	8	19	7	1	1
female	8	27	7	5	1
female	8	17	7	3	5
female	8	22	7	6	3
male	9	17	3	2	6
male	9	20	2	2	7
male	9	25	7	6	0
male	9	25	7	1	0
male	9	25	3	1	1

male	9	26	1	2	2
male	9	14	5	2	0
male	9	16	7	7	7
male	9	20	1	2	7
male	9	21	3	1	7
male	9	20	7	3	1
male	9	27	1	1	7
female	9	17	3	2	1
female	9	18	7	3	0
female	9	22	7	3	6
female	9	22	5	7	1
female	9	19	7	5	0
female	6	26	1	1	2
female	6	18	5	3	2
female	6	14	1	1	2

female	6	19	2	2	7
female	6	13	2	2	0
female	6	21	7	0	7
male	6	21	0	0	0
male	6	27	0	0	7
female	7	19	7	0	6
male	7	17	0	0	2
male	7	25	0	0	0
male	7	19	2	1	0
female	7	20	4	0	6
female	7	30	1	0	4
male	8	16	7	7	0
male	8	19	0	0	2
female	8	29	6	0	0
female	8	26	6	0	0

male	8	22	1	2	3
female	8	19	7	4	4
male	8	36	3	2	3
male	8	19	1	0	2
male	8	36	7	2	2
male	8	25	5	0	1
male	8	21	2	1	7
female	8	18	0	0	7
female	8	20	0	0	3
female	8	15	0	0	7
male	8	30	2	7	0
female	8	34	0	0	7
male	9	22	2	2	7
male	9	22	0	0	0
male	9	17	0	0	0

female	9	16	5	4	7
female	9	17	7	4	7
female	9	23	6	4	7
female	9	15	7	7	7
female	6	33	7	7	3
female	6	16	7	7	3
female	6	21	7	7	3
male	6	36	7	7	0
male	6	21	0	0	6
male	6	19	7	2	5
male	6	18	4	2	0
male	6	19	7	2	0
female	7	21	2	3	0
female	7	19	2	4	4
female	7	17	6	3	4

male	7	32	5	5	6
male	7	34	3	2	1
male	7	31	7	5	5
female	7	21	3	2	3
female	7	18	4	1	4
male	7	15	2	2	0
male	8	22	2	3	7
male	8	17	4	1	2
female	8	19	2	2	0
female	8	16	3	2	2
female	8	28	3	2	2
male	8	24	0	3	7
male	8	24	1	5	7
male	8	18	1	0	7
male	8	32	6	1	0

male	8	24	6	0	0
male	8	16	0	0	0
female	8	17	0	0	3
female	8	23	0	0	7
female	9	15	0	0	0
male	9	19	0	0	7
male	9	22	4	2	3
male	9	26	2	3	3
female	9	26	7	7	1
female	9	42	7	7	5
female	9	33	2	0	5
female	9	12	5	5	7
female	9	27	1	0	0
male	9	21	3	4	1
male	9	28	0	3	2

male	6	22	2	3	3
female	6	20	2	2	1
male	6	21	5	5	7
female	6	27	1	1	7
male	6	17	0	0	0
male	7	26	7	6	7
male	7	46	7	7	7
female	7	20	6	4	7
female	7	20	7	3	7
male	7	19	0	0	7
female	7	29	3	1	2
male	7	24	1	0	0
male	7	15	2	0	0
female	7	22	0	1	0
male	8	35	0	1	7

female	8	38	3	0	1
male	8	28	2	3	3
female	8	21	1	2	7
male	8	21	0	0	7
male	8	23	4	4	7
female	9	24	5	4	4
male	9	18	7	7	5
female	6	19	2	3	7
male	6	18	7	0	6
female	6	19	7	7	0
female	6	18	2	1	5
male	6	33	5	3	6
male	7	18	7	0	5
female	7	25	7	2	0
female	7	22	3	2	0

male	7	18	1	5	7
female	7	20	3	2	7
male	7	20	5	3	7
male	7	22	7	7	7
female	8	21	3	0	2
male	8	22	3	2	2
female	9	21	1	0	3
female	9	21	7	0	2
male	9	17	6	7	6
female	9	20	4	0	2
male	9	23	5	7	7
male	9	23	5	7	7
male	9	24	6	7	1
female	9	25	1	2	1
female	9	21	3	2	1

female	9	19	3	2	1
male	6	18	3	3	4
male	6	15	4	6	3
male	6	16	3	5	6
female	6	31	2	5	3
female	6	33	2	3	2
female	6	33	0	0	1
male	7	24	7	2	0
male	7	13	5	3	0
male	7	12	7	4	0
female	8	20	5	3	2
female	8	22	5	3	2
female	8	22	5	3	2
male	8	20	2	2	4
male	8	21	7	2	7

male	8	25	7	7	7
male	9	19	0	2	0
male	9	18	0	2	1
male	9	22	0	2	4
female	9	19	4	0	3
female	9	15	2	0	4
female	9	23	2	0	3
female	6	29	4	0	1
female	6	42	2	0	2
male	7	34	1	2	7
male	7	20	0	7	0
male	7	34	0	3	0
female	7	22	1	2	1
male	7	29	7	0	5
female	7	23	3	4	7

male	7	37	2	0	2
male	7	32	2	0	2
female	7	16	4	0	7
male	8	24	3	5	2
male	8	30	4	6	3
male	8	21	2	5	2
female	9	22	3	3	7
female	9	20	3	3	7
female	9	11	0	0	7
female	9	25	7	0	0
male	9	22	5	3	7
male	9	18	2	1	2
female	9	24	0	0	0
female	9	24	1	0	0

male	10	16	2	0	0
male	10	10	2	0	0
female	10	19	5	5	4
female	10	26	5	3	5
female	10	27	4	3	7
female	11	28	1	1	7
female	11	29	1	1	2
female	11	33	1	1	2
male	11	17	1	4	0
male	11	17	2	1	0
male	11	26	2	5	2
male	10	12	7	4	7
female	10	18	6	6	7
female	10	17	6	6	0
female	10	17	7	7	7

female	10	24	7	7	7
male	11	23	5	7	1
male	11	22	0	0	7
male	11	25	7	0	7
female	11	22	2	4	3
female	11	29	5	0	2
female	11	19	7	5	6
female	11	16	7	2	7
male	12	21	5	7	1
male	10	20	7	5	5
female	10	21	2	0	1
female	10	20	3	0	4
female	10	17	2	2	2
female	10	25	3	2	2
female	10	20	2	0	1

male	10	19	5	1	3
male	10	14	2	1	1
female	11	16	0	0	2
female	11	16	3	1	0
female	11	17	3	1	4
female	11	18	5	1	3
male	11	21	2	0	0
male	11	27	1	0	5
male	11	17	1	0	1
female	11	16	0	0	4
female	11	41	3	2	1
female	12	19	2	1	3
female	12	15	4	0	5
female	12	15	2	0	5
female	12	15	3	0	6

male	12	19	5	3	6
male	12	15	4	2	6
male	12	18	4	2	6
male	13	15	1	0	3
female	13	23	3	2	7
female	13	20	2	1	3
male	13	18	5	3	2
male	13	18	5	5	3
female	10	17	0	3	2
male	10	20	7	7	0
male	10	21	0	3	2
female	10	20	3	3	0
male	10	28	7	4	0
male	10	24	3	1	1
male	10	19	2	2	2

male	10	25	1	1	1
male	11	19	3	1	7
female	11	19	2	2	2
male	11	18	0	0	0
female	11	16	7	0	0
female	11	30	7	7	2
male	11	9	4	0	5
male	10	21	7	0	7
female	10	20	7	3	6
female	10	26	1	2	3
female	10	22	7	2	7
male	10	18	7	0	7
male	10	23	7	7	0
male	10	27	6	2	7
female	11	23	7	0	7

female	11	19	3	0	3
male	11	17	7	1	7
female	11	16	2	0	6
female	10	10	2	2	7
male	10	19	0	0	0
male	10	21	0	0	7
male	10	15	6	5	7
female	10	23	3	4	6
female	10	23	4	3	3
female	11	28	4	0	7
male	11	21	7	4	2
male	11	23	7	2	3
male	11	22	4	3	1
male	11	18	0	3	0
male	11	20	7	0	0

female	10	17	0	0	7
male	10	22	1	4	2
female	10	19	1	0	5
female	10	17	1	0	3
male	11	20	0	0	7
female	11	10	2	5	2
female	11	12	3	5	2
female	11	16	3	4	3
female	11	19	2	3	2
female	11	16	3	1	0
female	11	16	7	0	0
male	11	19	1	2	1
female	12	18	7	3	5
female	12	23	7	3	5
male	12	24	4	5	0

female	12	23	5	4	0
male	13	32	7	0	7
female	13	12	5	2	2
female	13	20	3	2	5
male	13	16	7	5	1
female	13	15	5	2	7
female	10	14	1	2	2
female	10	17	1	3	2
female	10	14	2	1	1
female	10	14	1	1	1
male	10	16	1	0	6
female	10	19	1	2	1
male	11	16	7	7	7
male	11	16	7	7	7
female	11	18	1	3	1

male	10	19	3	3	3
male	10	11	2	0	1
female	10	24	2	1	4
female	10	23	2	3	3
female	10	23	2	7	7
male	10	16	5	5	3
male	10	13	5	5	0
male	10	18	3	2	2
male	10	16	5	1	2
female	10	19	3	2	3
female	10	16	4	2	2
female	10	16	2	1	1
female	11	24	6	1	2
female	11	23	5	1	2
male	11	20	3	2	2

female	11	15	4	4	3
male	11	19	5	6	3
male	11	17	4	5	7
male	11	21	5	5	6
female	11	19	2	1	5
female	11	18	4	1	5
female	11	21	2	1	5
male	12	29	6	6	2
female	12	26	5	5	3
female	12	24	1	0	7
male	12	17	3	2	7
male	12	20	3	2	7
female	12	14	6	6	6
female	12	16	1	0	1
male	12	18	3	2	7

female	13	24	7	7	0
female	13	24	2	3	4
male	13	23	3	1	5
male	13	19	6	6	0
male	13	19	5	5	0
male	13	26	3	3	7
female	13	23	1	0	3
female	10	17	3	2	3
male	10	21	1	1	2
male	10	18	2	1	3
female	10	18	4	2	7
female	10	15	2	2	3
male	10	16	0	1	0
male	11	22	5	3	7
male	11	21	3	0	3

female	11	25	3	0	7
male	11	19	6	0	7
female	11	19	4	2	4
female	10	17	3	2	3
male	10	21	1	1	2
male	10	18	2	1	3
female	10	18	4	2	7
female	10	15	2	2	3
male	10	16	0	1	0
male	11	22	5	3	7
male	11	21	3	0	3
female	11	25	3	0	7
male	11	19	6	0	7
female	11	19	4	2	4
female	12	28	2	0	7

female	12	21	2	1	7
female	12	16	2	1	0
female	12	25	3	2	0
female	12	25	4	1	0
female	12	17	5	5	1
male	12	21	4	1	2
male	12	19	5	0	2
male	12	19	4	6	2
male	13	21	2	2	3
female	13	29	2	2	0
female	13	30	3	3	0
female	13	20	6	3	3
male	13	21	2	2	1
male	13	21	3	2	1
male	13	20	4	2	2

male	13	30	4	2	2
male	13	20	2	0	4
female	13	21	5	0	7
female	12	18	7	5	2
male	12	23	7	2	7
female	12	20	7	5	2
female	12	22	1	0	1
male	12	21	7	7	4
female	13	18	3	0	7
male	13	19	7	7	0
male	13	21	3	4	3
male	13	18	7	4	5
female	13	19	0	1	7
male	13	13	1	1	1
male	13	19	3	3	2

female	10	21	3	1	7
female	10	18	5	1	1
female	10	24	2	1	7
female	10	23	1	5	7
male	10	14	4	3	7
male	10	14	1	3	2
male	10	18	0	0	0
male	10	17	7	0	0
female	10	21	2	0	7
male	11	18	4	3	4
male	11	20	5	0	1
female	11	20	7	7	4
male	11	19	3	7	4
female	11	19	7	6	4
female	11	15	3	2	1

male	11	19	7	2	5
male	11	17	7	6	2
male	11	22	7	5	0
female	12	20	4	3	1
female	12	31	4	3	1
female	12	26	5	2	0
female	12	22	3	2	3
male	12	19	3	5	7
male	12	26	3	5	7
male	12	28	2	4	7
male	12	27	2	0	2
male	12	25	5	4	2
male	12	24	2	4	1
male	13	29	3	2	7
female	13	40	5	2	0

female	13	25	5	4	0
female	13	24	5	0	0
female	13	22	0	1	7
female	13	24	1	0	7
male	13	27	3	3	5
male	13	30	2	3	4
male	13	29	5	5	3
male	13	29	3	3	0
male	13	23	3	6	7
female	13	39	0	0	5
female	13	21	1	0	7
female	13	31	1	0	1
female	13	23	2	2	1
female	13	22	2	0	7
male	13	24	2	0	0

male	13	31	6	3	4
male	13	31	5	4	3
male	13	29	7	7	7
male	13	23	6	3	7
male	13	30	5	4	3
female	13	15	3	0	7
female	10	23	2	0	7
female	10	21	2	1	7
female	10	24	0	0	7
female	10	31	5	5	7
female	10	22	3	2	7
female	10	27	7	7	7
male	10	22	7	7	7
male	10	19	3	1	7
male	10	28	6	5	7

male	10	27	5	3	7
male	11	23	5	2	3
male	11	17	4	3	2
male	11	16	7	7	5
female	11	21	3	7	1
female	11	31	3	7	0
male	11	22	5	4	0
female	11	26	4	4	1
female	11	20	7	7	7
male	11	31	2	0	2
male	11	30	7	5	4
female	11	18	3	3	2
male	12	19	1	0	0
female	12	25	1	0	0
female	12	27	1	0	0

male	12	19	2	1	3
female	12	19	3	0	0
male	12	19	2	2	6
female	12	20	2	1	1
female	12	33	1	0	3
male	12	35	1	0	6
male	12	21	7	2	6
male	12	31	3	2	4
male	12	20	3	2	4
male	12	21	4	4	0
male	12	34	5	0	7
female	13	27	7	2	0
male	13	21	4	4	0
male	13	19	4	4	0
female	13	12	4	2	3

female	13	12	2	1	3
female	13	12	2	1	0
female	13	12	3	2	0
male	13	25	7	2	2
male	13	21	7	2	3
male	13	16	7	5	7
male	12	25	7	3	7
male	12	21	3	2	1
male	12	21	1	5	3
male	12	26	3	7	3
male	12	26	4	7	5
male	12	20	5	1	2
male	12	20	3	3	3
female	12	21	1	0	6
female	12	17	7	7	7

female	12	22	5	1	3
female	12	18	7	1	0
female	12	18	7	7	7
female	12	20	3	1	6
female	12	18	4	3	6
female	12	21	1	0	2
male	13	20	7	7	0
female	13	24	7	3	0
female	13	19	1	2	0
male	13	23	5	3	0
male	13	24	2	4	0
male	13	23	7	6	2
male	13	22	0	2	2
male	13	24	3	5	3
male	13	18	6	3	0

male	13	26	6	1	2
male	10	36	6	4	7
male	10	20	7	7	3
male	10	22	1	4	5
male	10	20	4	2	2
male	10	27	4	3	4
female	10	22	0	0	0
female	10	26	2	0	0
male	11	23	2	4	6
female	11	18	7	7	7
female	11	24	4	1	7
male	11	22	5	4	0
male	11	24	0	0	7
female	11	27	5	3	0
female	11	36	1	0	7

female	11	24	7	7	3
female	11	29	4	7	2
female	11	19	2	0	0
male	11	24	7	1	7
female	11	36	3	4	7
male	11	13	6	3	1
male	11	22	3	1	1
male	11	22	6	2	7
male	11	20	3	1	0
male	11	24	1	1	0
male	12	36	3	0	3
male	12	33	3	0	2
male	12	30	7	7	4
male	12	28	6	2	5
male	12	29	7	7	3

male	12	33	7	0	0
female	12	37	6	2	2
female	12	40	4	2	2
female	12	36	4	2	1
female	13	31	7	7	7
female	12	22	1	1	1
female	12	24	1	1	7
female	12	27	7	3	3
male	12	19	4	3	4
male	12	21	6	3	1
male	12	28	4	2	1
male	12	19	5	5	5
female	12	35	4	7	7
female	12	19	3	2	7
male	13	26	1	0	2

male	13	25	7	7	7
male	13	28	7	7	3
male	13	26	4	3	7
male	13	23	4	0	7
female	13	26	3	4	4
female	13	23	7	5	6
female	13	23	3	1	5
female	13	27	4	1	7
female	12	36	2	0	0
male	12	23	5	3	3
male	12	22	6	2	2
male	12	23	3	0	7
male	12	35	5	0	7
female	12	24	0	0	0
female	12	22	0	0	0

female	12	18	3	0	0
female	12	23	0	0	2
female	13	23	4	2	0
female	13	33	5	7	7
female	13	21	3	4	0
female	13	22	3	1	0
female	13	19	5	3	0
male	13	24	3	4	0
male	13	19	5	6	5
female	13	20	3	2	0
male	10	31	3	6	1
male	10	17	4	4	0
female	10	43	0	3	6
female	10	23	5	5	7
female	10	36	3	0	7

male	10	18	5	5	2
female	10	22	3	0	6
male	10	27	7	4	7
female	10	29	7	0	0
male	10	28	3	3	7
male	11	38	2	1	1
male	11	31	3	0	0
male	11	16	5	3	0
male	11	36	7	2	1
male	11	17	7	2	7
male	11	20	7	2	1
female	11	23	1	2	7
female	11	30	1	3	4
female	11	23	1	0	7
female	11	19	5	1	2

female	11	26	3	1	5
male	11	31	5	3	0
male	10	38	5	7	0
male	10	28	1	0	0
male	10	29	0	2	5
female	10	26	1	6	5
male	10	23	0	0	0
female	10	20	7	0	0
female	11	24	0	0	5
male	11	35	7	7	0
female	11	41	1	6	6
male	11	28	7	0	2
male	11	26	7	7	0
female	11	24	0	1	7
female	11	19	0	0	7

male	12	27	2	2	0
female	12	25	0	0	7
male	12	19	3	0	1
male	12	23	7	7	0
male	10	17	5	0	0
female	10	28	3	1	7
female	10	21	4	1	2
female	10	17	7	5	0
female	10	18	7	5	0
male	10	32	4	1	0
male	11	20	7	6	3
female	11	19	5	6	4
female	11	28	2	1	5
male	11	39	7	5	2
male	11	42	2	5	3

female	10	28	3	1	6
female	10	29	5	1	6
male	10	34	2	0	2
female	10	31	1	0	2
male	10	31	4	4	1
male	10	30	2	3	6
male	11	34	1	0	0
male	11	30	3	0	3
male	11	19	5	0	0
female	11	14	1	1	2
female	11	21	1	1	2
female	11	20	1	0	2
female	12	23	2	1	3
female	12	27	3	2	5
female	12	27	3	2	1

female	12	15	1	3	1
male	12	25	3	1	0
female	12	23	1	3	1
male	12	34	3	4	3
male	12	19	2	7	2
male	12	16	3	2	0
female	13	21	4	0	0
female	13	39	4	0	0
male	13	26	3	2	4
male	13	20	3	2	1
male	13	22	1	1	2
male	10	24	7	1	2
male	11	22	7	0	2
male	11	25	4	5	1
female	11	28	0	0	2

male	11	20	7	5	7
male	11	26	7	5	4
male	11	21	7	7	5
female	11	39	2	0	1
female	11	39	1	0	7
male	12	29	5	5	4
male	12	30	7	5	0
female	14	21	5	1	0
male	14	23	3	7	1
male	14	18	3	3	1
male	14	14	3	7	1
male	14	28	3	5	1
male	15	20	2	2	1
male	14	22	7	7	7

female	14	24	2	4	0
female	14	18	0	5	7
female	14	22	5	5	6
female	16	25	3	1	5
female	16	23	5	5	7
female	16	29	5	4	2
male	17	19	3	1	3
male	17	21	3	1	0
male	17	26	7	4	0
male	17	15	7	4	0
female	17	20	2	2	3
female	17	24	2	2	7
female	17	18	1	2	7
male	14	17	2	2	1
male	14	27	2	0	2

male	14	16	4	3	3
female	14	20	3	2	7
female	14	20	4	3	7
female	14	17	3	3	7
male	14	21	3	1	7
female	14	22	3	2	7
female	16	23	2	7	3
male	16	23	7	7	7
female	16	19	2	3	1
male	16	23	7	7	7
female	16	18	2	2	7
female	16	20	2	7	0
male	16	22	1	0	0
male	16	22	4	4	3
male	16	25	4	5	0

male	16	29	7	7	0
male	16	16	7	7	0
female	16	37	3	3	3
female	16	24	3	3	3
female	16	21	2	1	4
female	16	21	2	1	4
female	17	23	0	0	2
female	17	24	2	4	7
female	17	22	3	2	1
female	17	38	7	3	7
male	17	26	5	2	2
female	17	23	7	4	7
male	17	28	3	2	7
male	17	18	1	0	4
male	14	19	1	0	4

male	14	30	5	3	1
male	14	24	5	0	7
female	14	23	3	1	0
female	14	24	6	6	7
male	14	25	7	6	2
male	14	30	2	0	5
male	14	22	2	1	4
female	14	20	1	0	7
female	14	38	3	2	5
female	14	24	1	0	7
female	14	25	2	1	7
male	15	17	3	2	4
female	15	24	3	2	0
male	15	23	2	0	2
male	15	25	4	4	2

male	16	24	3	1	6
female	16	23	1	0	3
female	16	20	3	3	7
female	16	22	3	7	5
female	16	17	5	2	0
female	16	21	5	2	0
male	16	30	7	7	3
male	16	21	7	3	2
male	16	24	2	1	6
male	16	20	2	2	4
male	16	23	1	0	4
male	17	30	7	5	7
female	17	20	1	1	5
female	17	21	1	2	2
male	17	30	2	0	7

male	17	21	3	2	5
female	17	31	6	1	2
female	17	16	7	1	0
female	17	15	7	1	2
male	17	20	5	0	1
male	14	14	7	2	2
female	14	23	2	1	4
female	14	20	2	1	4
male	15	20	4	4	0
male	15	17	4	4	0
female	16	19	3	5	7
female	16	24	4	4	7
male	16	17	3	4	2
female	16	17	3	5	7
male	16	17	3	3	2

male	16	20	5	5	1
female	16	22	7	0	0
female	16	14	0	2	3
male	17	23	1	1	0
male	17	24	4	3	2
male	17	19	5	6	0
male	17	24	7	0	0
male	17	24	4	3	0
male	17	23	7	7	0
male	17	27	3	4	0
female	17	21	5	2	3
female	17	25	0	0	7
female	17	25	5	0	7
female	14	25	3	1	1
female	14	15	1	1	3

female	14	24	3	1	0
female	14	35	2	0	0
male	14	19	4	2	4
male	14	24	2	1	1
male	14	26	4	3	6
male	14	23	4	2	6
male	14	19	7	7	7
male	14	19	1	1	2
female	14	24	2	1	0
female	15	31	3	0	7
male	15	20	4	3	2
female	15	20	4	2	0
male	15	16	5	0	3
male	15	24	4	3	0
male	15	27	5	7	0

female	15	22	3	0	0
male	15	22	5	6	5
female	15	20	0	0	0
female	15	16	2	0	0
female	15	21	5	7	0
male	15	16	7	4	1
male	16	32	5	2	0
male	16	38	5	1	0
female	16	28	1	0	0
female	16	22	3	2	3
female	16	21	1	0	1
male	16	22	5	3	2
female	16	28	1	0	0
male	16	22	4	1	0
male	16	25	5	1	0

female	16	34	1	0	0
male	16	26	4	1	0
female	16	29	2	0	0
male	17	24	1	0	0
male	17	25	7	5	4
male	17	20	1	1	0
male	17	30	7	0	4
female	17	22	0	1	1
male	17	21	2	1	4
male	18	22	3	1	4
female	18	18	3	0	7
female	14	30	3	4	7
male	14	11	1	0	2
female	14	28	1	0	1
female	14	20	3	6	4

female	14	29	3	5	4
male	14	20	1	0	2
female	14	20	3	5	4
male	14	20	0	1	1
female	14	22	1	2	7
female	14	20	1	0	2
male	14	15	2	1	7
male	14	14	5	1	7
male	14	18	5	7	0
male	14	39	7	3	7
female	15	20	1	0	7
female	15	20	2	7	7
male	15	20	6	6	3
male	15	37	7	7	3
male	15	16	3	3	7

male	15	22	1	1	5
male	15	18	1	1	7
female	16	25	1	0	2
female	16	27	1	1	2
female	16	30	5	2	1
female	16	22	4	1	2
female	16	31	4	0	1
female	16	34	7	1	1
female	16	24	4	1	2
male	16	24	4	4	2
male	16	28	3	1	4
male	16	38	3	0	0
male	16	32	1	2	3
female	17	29	1	0	0
female	17	23	0	0	2

female	17	22	0	0	2
male	17	15	4	3	7
male	17	25	7	7	2
male	17	27	4	3	7
male	14	26	3	5	1
female	14	32	5	3	1
female	14	25	2	0	2
female	14	23	3	2	0
female	14	30	3	2	4
male	14	20	3	1	7
male	14	36	7	5	7
male	14	46	2	1	7
male	14	38	3	2	7
male	14	31	6	5	3
male	15	31	5	2	7

male	15	22	6	1	7
male	15	13	7	7	2
male	15	29	7	7	7
female	15	29	7	6	4
female	15	21	3	0	2
male	15	36	7	4	2
female	15	22	3	0	4
female	15	23	2	2	1
female	15	28	3	1	0
male	15	39	7	7	0
male	16	24	7	2	0
male	16	28	7	5	3
male	16	41	6	5	3
male	16	32	1	0	0
male	16	17	2	0	0

male	16	31	7	5	2
male	16	21	3	1	1
male	16	16	1	1	0
male	16	22	0	0	0
male	16	22	2	2	0
female	16	34	1	4	7
female	16	21	1	5	7
female	16	18	2	1	4
female	16	20	4	6	3
female	16	21	5	5	2
female	16	24	1	0	4
female	16	13	0	0	6
female	17	21	1	0	0
male	17	24	2	2	0
male	17	27	0	0	2

female	17	16	0	2	0
male	17	23	7	2	4
male	17	32	5	2	7
male	17	33	2	0	2
female	17	33	1	0	0
female	17	25	2	1	0
male	17	13	2	1	7
male	17	26	0	1	4
female	17	32	1	5	7
female	17	33	1	5	7
female	17	19	1	5	2
female	17	25	2	3	7
female	17	20	7	5	2
female	17	23	3	3	2
female	17	28	2	2	0

female	14	23	5	1	0
male	14	15	4	5	3
male	14	19	5	5	3
male	14	20	3	2	2
female	14	29	0	0	0
female	14	19	7	7	7
female	14	32	7	7	2
female	14	17	2	1	0
male	14	22	7	4	3
male	14	23	3	2	7
male	15	29	2	0	0
male	15	17	2	0	0
male	15	39	2	0	0
male	15	20	2	0	0
male	15	16	2	0	0

male	15	19	1	0	0
male	15	19	1	0	0
female	15	31	5	1	0
female	15	26	5	2	7
male	15	25	7	7	3
male	15	24	2	2	0
male	15	26	7	3	2
male	15	28	7	2	0
male	15	17	3	4	6
male	15	30	4	2	3
male	15	19	5	5	0
female	15	25	3	2	7
female	15	27	1	0	2
female	15	23	1	0	4
female	15	24	1	4	5

female	15	23	5	1	1
female	16	31	5	1	4
female	16	30	4	2	3
female	16	17	2	2	7
female	16	34	5	1	3
female	16	24	6	2	2
male	16	15	4	3	7
male	16	27	7	7	7
male	16	18	2	1	5
male	16	25	7	7	4
male	16	20	5	5	7
male	16	17	2	0	7
male	16	44	1	0	7
male	16	32	1	0	3
male	17	28	5	3	2

female	17	25	4	2	0
female	17	23	1	1	7
female	17	20	7	1	7
female	17	16	1	1	7
female	17	19	1	0	6
female	17	21	3	1	7
female	17	15	3	0	1
female	17	23	2	0	0
female	17	22	1	0	0
male	17	20	7	7	7
male	17	24	7	7	0
male	17	26	7	4	1
female	17	21	2	1	7
female	17	24	3	0	0
male	14	31	1	1	0

male	14	26	3	0	2
male	14	23	1	0	0
female	14	30	7	7	5
female	14	26	4	2	2
female	14	27	5	2	2
female	14	21	3	3	2
female	14	37	4	4	2
female	14	23	3	2	5
male	15	22	6	4	6
female	15	25	2	1	2
female	15	27	2	5	0
female	15	30	4	1	2
female	15	29	4	5	0
male	15	18	5	2	3
female	15	29	5	2	5

female	15	23	3	2	6
male	15	37	7	7	0
male	15	29	6	6	5
male	15	24	5	2	2
male	15	34	1	0	3
female	15	23	2	2	6
female	15	23	7	7	7
female	16	43	4	4	0
female	16	38	5	5	0
male	16	35	3	2	5
male	16	29	6	5	7
female	16	22	2	0	7
female	16	24	0	0	1
female	16	29	0	0	7
female	16	20	4	0	3

female	16	23	5	1	0
male	17	19	3	1	0
male	17	26	4	1	0
male	17	44	6	2	0
female	17	63	1	0	0
female	17	29	3	2	3
female	17	27	2	0	7
male	17	28	7	6	0
male	14	27	4	0	7
male	14	21	2	1	3
female	14	19	4	1	7
female	14	32	6	2	6
female	14	17	3	0	7
female	14	42	4	1	7
female	14	37	1	0	0

female	14	23	3	1	4
male	15	42	2	2	7
male	15	25	0	0	3
male	15	18	2	0	4
male	15	29	3	3	4
male	15	26	4	4	3
male	15	27	5	1	7
female	15	37	3	0	2
female	15	22	7	0	0
female	15	23	2	0	3
female	15	21	2	3	2
male	15	36	7	0	7
female	16	29	1	5	7
female	16	35	1	6	2
female	16	34	2	0	5

female	16	29	1	0	6
male	16	35	1	1	4
male	16	16	3	2	2
male	16	31	4	1	3
male	16	38	2	5	0
male	16	37	1	0	5
male	16	49	5	3	7
female	16	21	7	5	7
female	17	27	7	1	0
female	17	21	4	1	0
male	17	27	7	7	0
female	17	28	3	1	0
male	17	50	5	2	4
female	17	35	7	7	7
female	17	46	3	4	2

female	17	38	1	5	4
female	14	21	2	1	0
female	14	26	3	2	0
female	14	25	1	0	0
male	14	28	2	0	0
male	14	27	2	1	0
male	14	26	2	1	0
female	15	22	2	3	0
male	15	26	3	1	0
female	15	26	2	1	0
male	15	29	3	3	1
male	15	26	2	1	0
female	15	24	2	2	0
male	15	25	3	2	0
male	15	34	2	0	0

male	15	34	3	1	3
male	15	42	7	7	0
female	15	24	3	0	7
male	16	46	7	7	7
male	16	48	4	3	3
male	16	49	5	5	7
male	16	69	4	2	3
male	16	29	5	3	3
male	16	25	4	3	3
male	16	28	2	5	3
male	16	23	5	3	0
male	16	27	5	0	2
female	17	19	0	2	6
male	17	34	5	7	0
female	17	28	1	1	0

female	17	24	0	0	0
female	17	21	0	0	0
female	17	32	3	3	0
male	17	25	3	7	4
male	17	31	5	5	3
male	17	32	7	3	2
male	17	27	2	2	0
male	17	27	3	2	3
female	14	27	7	2	5
female	14	19	7	3	4
male	14	23	7	5	2
male	14	23	7	6	3
male	14	25	7	5	4
female	14	39	4	0	0
female	14	27	7	2	0

female	15	24	5	3	7
male	15	23	1	1	1
female	15	33	3	1	2
female	16	19	2	2	2
female	16	21	3	2	4
male	16	19	3	4	2
male	16	18	4	4	7
female	16	40	4	1	7
male	17	33	6	5	5
female	17	40	5	3	2
female	17	24	3	2	7
female	17	22	3	3	7
